# DBDNMF: A Dual Branch Deep Neural Matrix Factorization method for drug response prediction

**DOI:** 10.1371/journal.pcbi.1012012

**Published:** 2024-04-04

**Authors:** Hui Liu, Feng Wang, Jian Yu, Yong Pan, Chaoju Gong, Liang Zhang, Lin Zhang

**Affiliations:** 1 School of Information and Control Engineering, China University of Mining and Technology, Xuzhou, Jiangsu, China; 2 Department of Ophthalmology, Xuzhou First People’s Hospital, Xuzhou, Jiangsu, China; 3 Department of Gastrointestinal Surgery, Xuzhou Central Hospital, Xuzhou, Jiangsu, China; University at Buffalo - The State University of New York, UNITED STATES

## Abstract

Anti-cancer response of cell lines to drugs is in urgent need for individualized precision medical decision-making in the era of precision medicine. Measurements with wet-experiments is time-consuming and expensive and it is almost impossible for wide ranges of application. The design of computational models that can precisely predict the responses between drugs and cell lines could provide a credible reference for further research. Existing methods of response prediction based on matrix factorization or neural networks have revealed that both linear or nonlinear latent characteristics are applicable and effective for the precise prediction of drug responses. However, the majority of them consider only linear or nonlinear relationships for drug response prediction. Herein, we propose a Dual Branch Deep Neural Matrix Factorization (DBDNMF) method to address the above-mentioned issues. DBDNMF learns the latent representation of drugs and cell lines through flexible inputs and reconstructs the partially observed matrix through a series of hidden neural network layers. Experimental results on the datasets of Cancer Cell Line Encyclopedia (CCLE) and Genomics of Drug Sensitivity in Cancer (GDSC) show that the accuracy of drug prediction exceeds state-of-the-art drug response prediction algorithms, demonstrating its reliability and stability. The hierarchical clustering results show that drugs with similar response levels tend to target similar signaling pathway, and cell lines coming from the same tissue subtype tend to share the same pattern of response, which are consistent with previously published studies.

## Author summary

As is known, the phenotype of cancer is closely related to gene expression in the human body. In the era of precision medicine, an increasing amount of anti-cancer drug response data is urgently needed for individualized therapy. Measurements with wet-experiments are time-consuming and costly, and computational methods are necessary. Existing methods mainly focused predominantly on linear or nonlinear relationships between cells and drugs for prediction. However, it has been shown that both linear and nonlinear relationships could contribute to more precise response prediction. Here, we propose a Dual Branch Deep Neural Matrix Factorization (DBDNMF) method to better use both linear and nonlinear relationships and the experimental results show that it outperforms state-of-the-art algorithms. Our results suggest that drugs showing similar response levels tend to target similar signaling pathways while cell lines sharing similar response patterns tend to come from the same tissue subtype. The analysis of experimental results may benefit medical decision-making.

## Introduction

In recent years, cancer has become a serious illness that endangers both life and health. The rates of morbidity and mortality are rising globally right now. Due to the heterogeneity of tumors, there are variations in medication sensitivity throughout tumor deterioration [1]. Based on the results of clinical experiments, precision medicine, which has become the dominant trend in medicine, asks for customized treatment strategies for individual cancer patient [2]. Numerous academic institutions and organizations have constructed cancer pharmacogenomics databases as high-throughput sequencing technology has advanced over time [3]. However, to recommend precise medications for each patient based on response, clinical trials must be conducted to measure their individual response, which is time-consuming and laborious. Prediction of unknown anti-cancer medication responses based on known ones has therefore emerged as a crucial area for further study in the bioinformatics community.

The matrix factorization-based drug response prediction method has demonstrated satisfactory prediction performance in several recent investigations. In MF-based methods, the response matrix is roughly determined by the product of latent drug and latent cell line variables. Therefore, MF approaches are widely used in response prediction due to their effectiveness in learning latent features. According to the MF concept, the response matrix ***R*** can be divided into the product of two or more low-rank factor matrices, as illustrated in (1).

R=C×D (1)

where the matrix ***R***∈ℝ^*m*×*n*^, the latent factors ***C***∈ℝ^*m*×*r*^, ***D***∈ℝ^*r*×*n*^, and *r* = *rank*(***R***) [4]. The unknown responses can be recovered by finding the best of ***C*** and ***D***. Numerous strategies have been put forth to improve the MF method’s accuracy. A prediction model based on kernel Bayesian matrix factorization (KBMF), which incorporates genomic information, drug chemical characteristics, and target information, was proposed by Ammad-ud-din et al. It has been demonstrated that this information is of practical importance for drug response prediction [5]. The chemical characteristics of drugs and data on cell line gene expression were used by Wang et al. to construct cell line similarity and drug similarity matrices, which were then combined with the conventional matrix decomposition model as regularization terms to accurately predict the drug-gene association [6]. Suphavilai et al. suggested a thorough model (CaDRReS) to pinpoint drug response mechanisms by utilizing information on how different medications interact with various cell types. To forecast unknown drug reactions, the model learns the projections of medicines and cell lines in a latent space [7]. To sparse the similarity matrix, Guan et al. presented weighted graph regularization matrix decomposition (WGRMF), which created p-nearest neighbor graphs of pharmaceuticals and cell lines, respectively. Experimental findings clearly show how effective this strategy is at forecasting drug responses [8]. Based on the idea of subspace clustering, Zhang et al. proposed a new self-expression matrix completion model (SEMCM) aimed at improving the prediction performance of drug response prediction [9]. Liu et al. proposed a Neural Matrix Factorization (NeuMF) framework, which used a deep neural network to figure out drug and cell lines’ latent variables to help predict the unknown responses of cell lines to drugs [10]. These MF-based methods have shown good predictive impact by providing linear reconstructions of the observed data. That means these MF-based methods depend on the linear latent variable models [11].

Deep neural networks’ success in recent years has been attributed to their efficiency in learning hidden characteristics and data representations. These techniques can also approximate the data from nonlinear latent variable models by utilizing a nonlinear activation function, as demonstrated in (2),

R=f(D)orR=f(C) (2)

where *f*(⋅) is a nonlinear transformation. By *f*(⋅), the latent factor ***D*** or ***C*** can approximately predict the unknown responses in ***R***. For instance, Hossein et al. proposed a multi-omics data integration model based on a deep neural network (MOLI) to predict the sensitivity of targeted drugs. MOLI combines three independent sub-networks for extracting cell line mutation, expression, and copy number change features, as well as four multi-layer feedforward sub-networks. The final sub-network uses the characteristics that the other three sub-networks produce as its input and then produces drug sensitivity [12]. Jia et al. developed a deep variational autoencoder (VAE) model to generate representative models using expression profiles and train prediction models for drug response based on the latent representation. It was shown that the VAE could correctly predict drug response and effectively manage the overfitting issue [13]. The vector Embedding neural network (VENN) predicts the drug response by generating a corresponding k-dimensional Embedding vector by the drug or cell line number, which is then used to represent k features of the drug or cell line. The Self-information Collaboration Neural Network (SCNN) extracts the drug and cell line information from the response matrix through a deep neural network. The information is projected into a latent space of the same dimension where they can interact to produce the predicted drug response. Graph neural networks have shown ground-breaking performance in the field of bioinformatics. Zhu et al. proposed a novel Drug response prediction framework comprised of Twin Graph neural networks for Drug Response Prediction (TGDRP) and a Similarity Augmentation (TGSA) module to fuse fine-grained and coarse-grained informatio [14]. Peng et al. proposed an end-to-end algorithm based on Multi-Omics Fusion and Graph Convolution Network (MOFGCN) to predict drug sensitivity in cell lines [15]. Peng et al. also proposed a neighborhood interaction-based heterogeneous graph convolution network method (NIHGCN) for anticancer drug response prediction [16]. These methods reveal that nonlinear latent characteristics are also applicable and effective for the precise prediction of drug response. As a result, both linear and nonlinear association features can contribute to the precise prediction of anti-drug responses. However, current solutions are limited since the majority of them only consider linear or nonlinear contributions.

To address the above issue, we propose here a Dual Branch based Deep Neural Matrix Factorization method (DBDNMF) to extract both linear and nonlinear contribution features for drug response prediction. Experiments are also carried out on both CCLE and GDSC datasets, and the results show that, compared with some state-of-the-art algorithms, DBDNMF method can achieve better prediction performance. This paper is organized as follows. Section 2 introduces the datasets and the proposed DBDNMF method. Section 3 evaluates the performance of DBDNMF based on both CCLE and GDSC datasets. Section 4 gives our conclusion.

### Experimental design

#### Datasets

The first dataset comes from Cancer Cell Line Encyclopedia (CCLE), which collected activity area to represent the degree of sensitivity of a given cell line to a given compound. The activity area refers to the area under the fitted dose-response curve for each experiment. The higher the activity area is, the more sensitive the cell line is to the drug. From this dataset, the response levels of 491 cell lines to 23 drugs are considered for further study. The sparsity of the CCLE matrix is 3.75% out of the 11293 entries, of which 10870 have been determined by a pharmacological method, while the rest 423 remain unknown.

The second dataset comes from Genomics of Drug Sensitivity in Cancer (GDSC) with the lasted version. It uses the half maximal inhibitory concentration (IC50) to represent cell line–drug interactions. IC50 indicates the drug response characteristics by natural logarithm means the concentration at which the compound reaches a 50% reduction in cell viability. The lower the IC50 is, the more sensitive the cell line is to the drug. From this dataset, the response levels of 969 cell lines to 295 drugs are considered, including 285855 entries, with 242036 entries known and 43819 to be predicted. The sparsity of the GDSC matrix is 15.32%, and the missing values are distributed in blocks, which brings more difficulties to prediction.

#### Performance evaluation

In this paper, 10-fold cross validation experiment is conducted for evaluation [17]. To be specific, the observed drug cell-line responses (known entries in ***R***) are first divided into 10 disjoint folds that have approximately the same number of instances randomly. Then each fold in turn plays the role for testing the model induced from the rest 9 folds. Then, a predictive matrix of the same size as the original can be recovered by combining the outcomes of 10 folds.

Pearson correlation coefficient (PCC) and Root mean square error (RMSE) are adopted to assess the prediction performance [18,19]. PCC can inspect the degree of correlation between the predicted value and the original value, while RMSE can calculate the error between the predicted value and the original value. On this basis, we further refine the evaluation indices to the averaged Pearson correlation coefficient (ave_PCC) defined in (3) and the Root mean squared drug error (ave_RMSE) in (4) from the perspective of real data application.

$$\mathrm{ave}\_\mathrm{PCC}=\frac{1}{m}\sum_{i=1}^{m}\frac{\sum_{j=1,(i,j\in \Omega )}^{n}\left(R_{i,j}-{\bar{R}}_{j})({\hat{R}}_{i,j}-{\bar{\hat{R}}}_{j}\right)}{\sqrt{\sum_{j=1,(i,j\in \Omega )}^{n}{\left(R_{i,j}-{\bar{R}}_{j}\right)}^{2}}\sqrt{\sum_{j=1,(i,j\in \Omega )}^{n}{\left({\hat{R}}_{i,j}-{\bar{\hat{R}}}_{j}\right)}^{2}}}$$ (3)

$$\mathrm{ave}\_\mathrm{RMSE}=\frac{1}{m}\sum_{i=1}^{m}\sqrt{\frac{\sum_{j=1,(i,j\in \Omega )}^{n}{\left(R_{i,j}-{\hat{R}}_{i,j}\right)}^{2}}{\left|{\hat{R}}_{i,j}\right|}}$$ (4)

where $\bar{R}$ and $\bar{\hat{R}}$represent the mean of ***R*** and $\hat{R}$ respectively. |***X***| represents the number of elements in ***X***. *m* is the number of drugs, and *n* is the number of cell lines.

Sensitive and resistant cell lines for each drug will be of greater assistance when choosing a drug therapy for cancer cell lines. In the experimental analysis, the response values of all cell lines for each drug are first divided into quartiles based on the response value, with the first and the fourth quartiles representing sensitive (resistant) and resistant (sensitive) ones respectively. Thus, ave_PCC_sr and ave_RMSE_sr are defined to indicate the averaged PCC and RMSE of these cell lines only.

### Experimental results

#### Global effect removal helps improve the prediction

**Fig 1:**
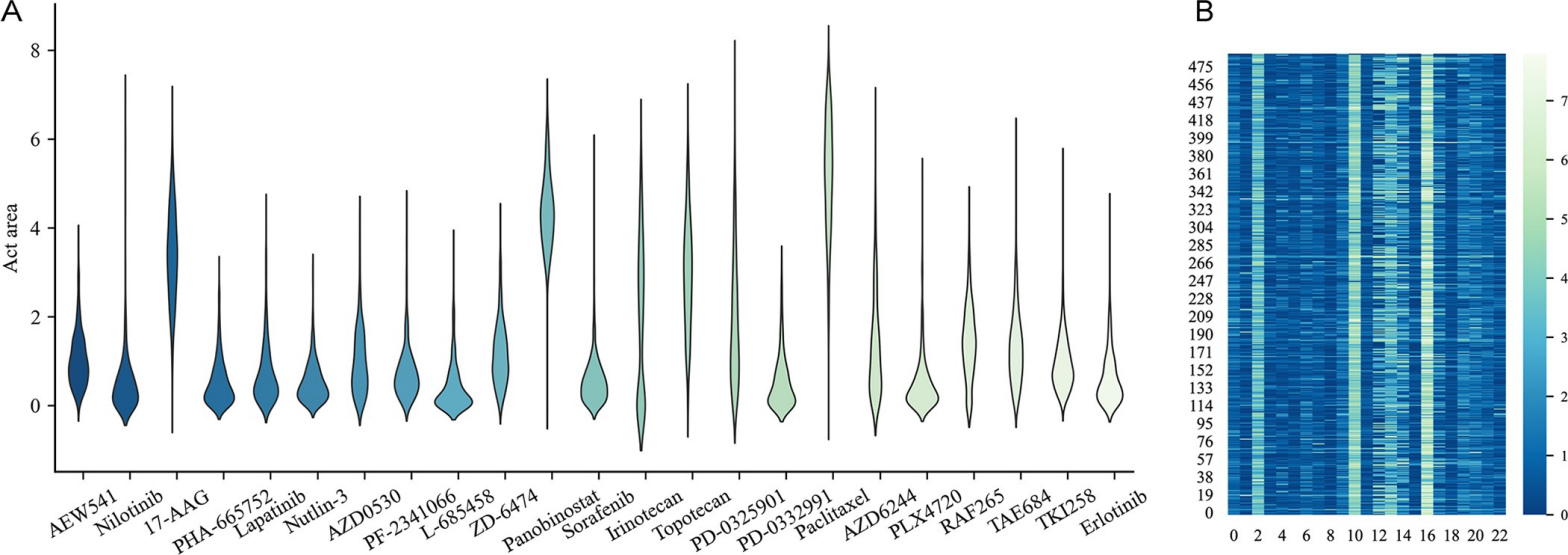
Distribution of CCLE cell line-drug response values. **A)** Violin-plot of existing values for 23 drugs in CCLE; **B)** Heatmap of cell lines-drug response matrix.

As shown in **Fig 1**, the distribution range of responses among different drugs in CCLE dataset is quite different, which is consistent with the fact that there are substantial variations in responses because different drugs differ in their molecular structure and chemical properties. At the same time, data deviation during collection is unavoidable. Reasonable variations will influence the results of the prediction, but a significant departure from the global benchmark will make the prediction difficult. Therefore, appropriate data preprocessing is expected to help to enhance the prediction.

In this paper, the matrix preprocessing method of removing Global Effect Removal (GER) is adopted, which was first proposed by Yehuda Koren et al. in the Netflix Grand Prize and applied to movie score prediction [20]. To eliminate the bias between the different drug responses, we preprocessed datasets so that the drug responses of all cell lines have the same benchmark.

Based on the normalization of global effect, the original responses can be considered as composed of global effect, drug-specific effect, cell line-specific effect, and interaction effect between drug and cell line, as shown in (5).

$$R=R_{global}+R_{drug}+R_{cell}+R_{res}$$ (5)

where ***R***_*global*_ is the global average of responses, which indicates that the unknown response depends on the benchmark of overall responses; ***R***_*drug*_, the average of each column in the response matrix, demonstrates that the unknown response influenced by the specific effect of cell lines; ***R***_*cell*_, the average of each row in the response matrix, suggests that the unknown response relies on the specific effect of drugs; ***R***_*res*_ is the residual after removal of the effect, which represents the interaction effect between the drug and the cell line. Compared with the prediction by the original responses, the residual after eliminating the above effects can improve the accuracy.

**Fig 2:**
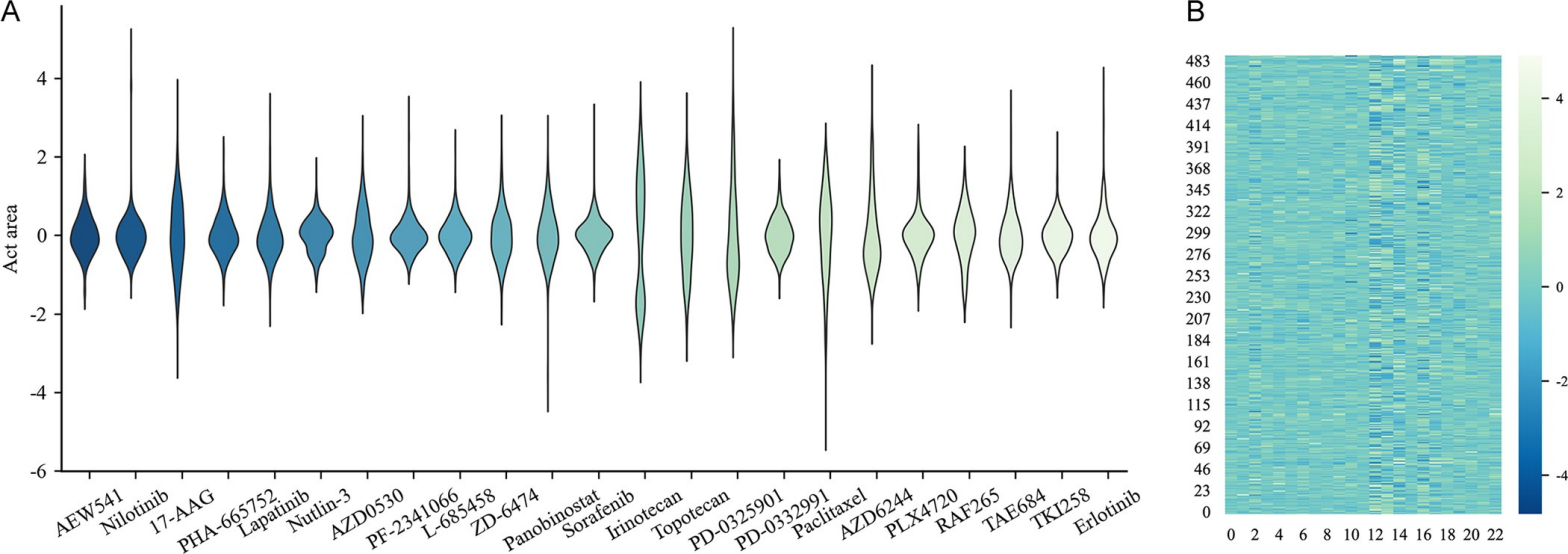
Distribution of CCLE cell line-drug response values after global effect removal. **A)** Violin-plot of 23 drugs’ response distribution in CCLE; **B)** Heatmap of cell line-drug response matrix.

**Fig 3:**
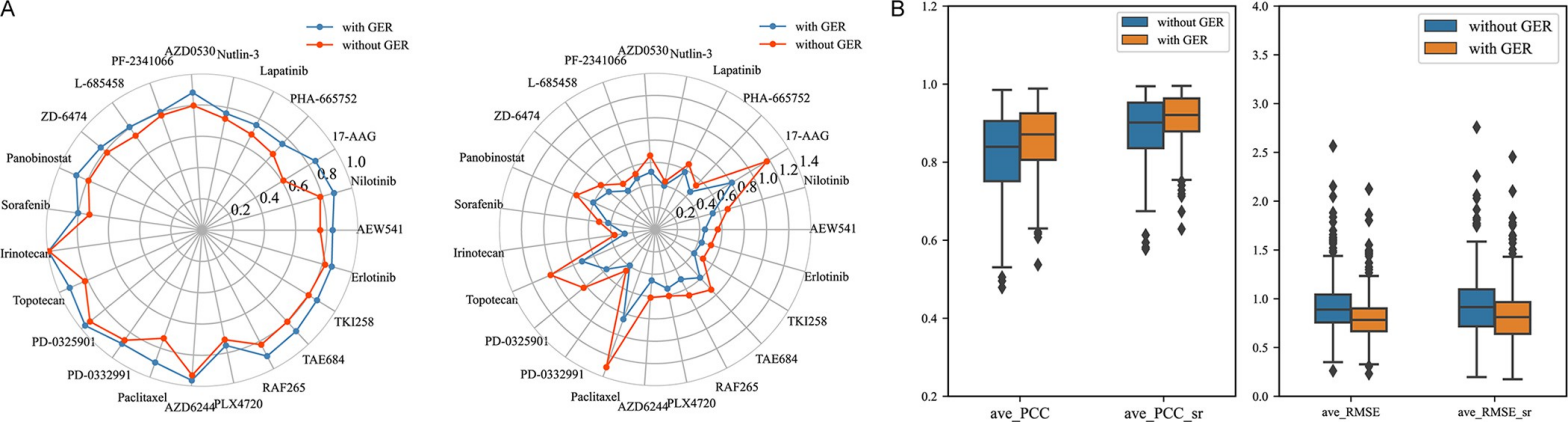
Comparison of results with and without GER preprocessing for prediction. **A)** Radar plots of PCC_sr and RMSE_sr for 23 drugs in CCLE; **B)** Box-plots of ave_PCC (ave_PCC_sr), ave_RMSE (ave_RMSE_sr) on the GDSC.

As shown in **Fig 2**, preprocessing not only causes a decrease in the overall value but also significantly lowers the deviation between columns and rows. 10-fold cross validation experiments are performed on the original matrix ***R*** and the matrix ***R***_*res*_ preprocessed by GER under the assumption that no model parameters are changed. According to **Fig 3**, the PCC_sr after preprocessing is higher than that without preprocessing, and the RMSE_sr is correspondingly lower in CCLE and GDSC datasets. Therefore, it has been demonstrated that GER preprocessing on CCLE and GDSC increases prediction accuracy.

From a mathematical perspective, GER guarantees the sensitivity/resistance of drug cell lines and also eliminates the significant difference between cell lines and drugs.

### Dual branch structure helps improve prediction

**Fig 4:**
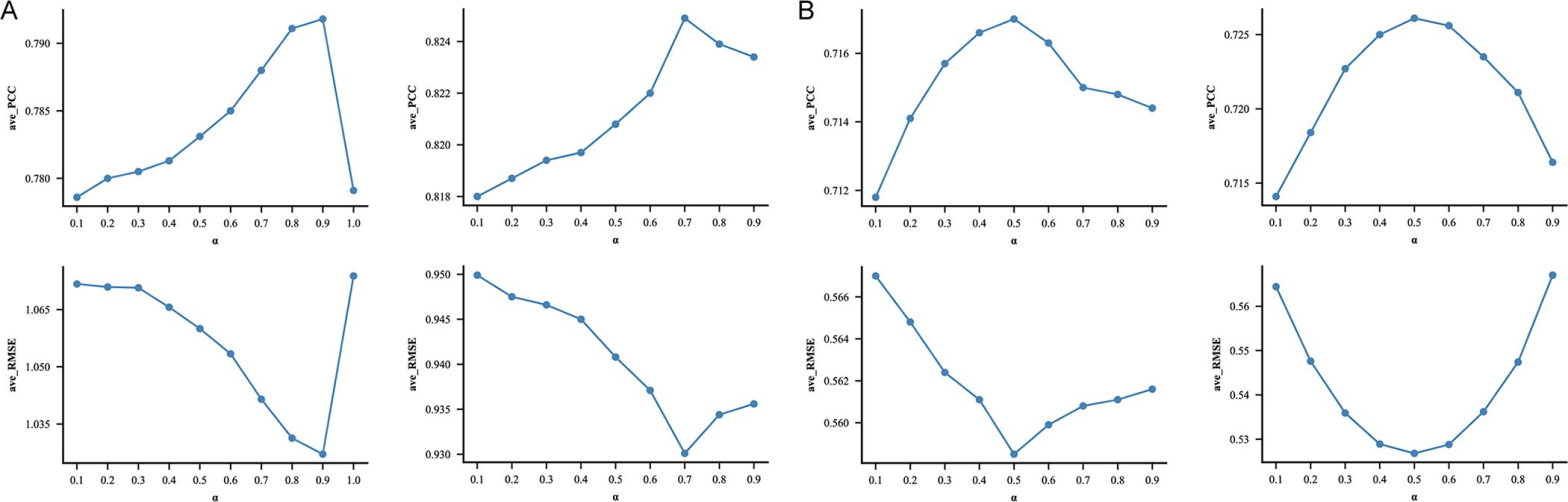
Effect of the weight of the nonlinear structure *α* on prediction. **A)** ave_PCC and ave_RMSE of network based on drug and cell line latent factors in GDSC; **B)** ave_PCC and ave_RMSE of network based on drug and cell line latent factors in CCLE.

This section discusses the role of parameters *α* in the model, namely the contribution of nonlinear structure in the network, and determines its optimal weight. The experiment is based on the network with drug factors as input and the network with cell line factors as input respectively. The other network parameters are left unaltered, setting *α* as [0.1, 0.2,…, 0.9] in turn. **Fig 4** records the results on CCLE and GDSC datasets by comparing the experimental results under various *α* with the ave_PCC and ave_RMSE obtained by 10-fold cross validation as indices.

**Fig 4A** records the network experimental results based on drug and cell line latent factors under various *α* in GDSC dataset. Combined with PCC and RMSE comprehensive evaluation, the best results for the model occur when *α* is 0.9 or 0.7, that is when nonlinear structure predominates in the network; The network experimental results based on drug and cell line latent factors under various *α* in CCLE dataset are shown in **Fig 4B**. When *α* is 0.5, that is, when nonlinear structure and linear structure are equally distributed in the network, the result of the model is best.

Consequently, the experimental results on two datasets are compared, and it is found that the weight varies depending on the dataset. For the two datasets in this paper, the prediction ability of linear structure in GDSC is inferior to that of the nonlinear structure; however, in CCLE, the nonlinear structure and linear structure achieve the best performance when they cooperate to form a network. The following conclusions are reached after taking into account the analysis of experimental findings: since GDSC dataset has a much larger scale and sparser matrix than CCLE dataset, it relies more on the nonlinear structure.

### DBDNMF improves prediction by both linear and nonlinear contributions

The outcomes of the 10-fold cross validation are compared to the leading methods, WGRMF, SRMF, KBMF, NeuMF, and TGSA which are considered state-of-the-art (SOTA). The experimental parameters of WGRMF, SRMF, KBMF, NeuMF, and TGSA are set according to the optimum. The CCLE and GDSC datasets are used to test the methods, and the outcomes are displayed in Tables **1** and **2**, respectively. The standard deviation of evaluation is shown in brackets in the table; DBDNMFGER denotes the DBDNMF prediction preprocessed by the Global Effect Removal.

**Table 1:** Comparison of ave_PCC (ave_PCC_sr), ave_RMSE (ave_RMSE_sr) of different methods for 10-fold cross-validation on CCLE dataset.

Methods	*ave_PCC_sr*	*ave_RMSE_sr*	*ave_PCC*	*ave_RMSE*
KBMF	0.65(±0.10)	0.81(±0.20)	0.61(±0.10)	0.64(±0.17)
SCNN	0.68(±0.07)	0.80(±0.49)	0.61(±0.12)	0.62(±1.18)
VENN	0.72(±0.10)	0.74(±0.57)	0.64(±0.11)	0.43(±0.44)
SRMF	0.78(±0.07)	0.74(±0.23)	0.71(±0.09)	0.57(±0.18)
WGRMF	0.79(±0.07)	0.69(±0.19)	0.72(±0.09)	0.56(±0.19)
NeuMF	0.78(±0.09)	0.68(±0.25)	0.71(±0.10)	0.57(±0.24)
DBDNMF	0.79(±0.09)	0.65(±0.16)	0.73(±0.10)	0.53(±0.22)
**DBDNMFGER**	**0.86(±0.07)**	**0.52(±0.13)**	**0.81(±0.08)**	**0.44(±0.10)**

**Table 2:** Comparison of ave_PCC (ave_PCC_sr), ave_RMSE (ave_RMSE_sr) of different methods for 10-fold cross-validation on GDSC dataset.

Methods	*ave_PCC_sr*	*ave_RMSE_sr*	*ave_PCC*	*ave_RMSE*
KBMF	0.50 (±0.15)	2.23(±0.66)	0.40(±0.14)	1.69(±0.50)
SCNN	0.63(±0.16)	1.58(±0.81)	0.53(±0.16)	1.26(±0.82)
VENN	0.63(±0.17)	1.49(±0.99)	0.53(±0.17)	1.08(±0.86)
SRMF	0.71(±0.15)	1.73(±0.46)	0.62(±0.16)	1.43(±0.36)
WGRMF	0.73(±0.14)	1.71(±0.16)	0.64(±0.16)	1.37(±0.15)
NeuMF	0.82(±0.09)	1.43(±0.48)	0.76(±0.10)	1.27(±0.82)
TGSA	0.85(±0.10)	1.01(±0.36)	0.78(±0.12)	0.88(±0.30)
DBDNMF	0.89(±0.08)	0.94(±0.35)	0.82(±0.10)	0.93(±0.30)
**DBDNMFGER**	**0.91(±0.06)**	**0.83(±0.31)**	**0.86(±0.08)**	**0.81(±0.25)**

According to **Table 1**, the DBDNMFGER proposed in this paper yields the highest value (0.81 and 0.86) in ave_PCC (ave_PCC_sr) and the lowest value (0.44 and 0.52) in ave_RMSE (ave_RMSE_sr). With regard to the GDSC dataset, our model is still able to predict the drug responses with the best performance in ave_PCC (ave_PCC_sr) and ave_RMSE (ave_RMSE_sr), as shown in **Table 2**. This demonstrates the robustness of learning and prediction capabilities of the neural matrix factorization with dual branch structure, particularly when applied to the datasets processed by Global Effect Removal. The model can fit the factor matrix of drugs and cell lines thanks to flexible input while achieving response prediction which is important for analyzing the latent correlation properties between cells and drugs.

### Individual drug prediction evaluation on CCLE

**Fig 5:**
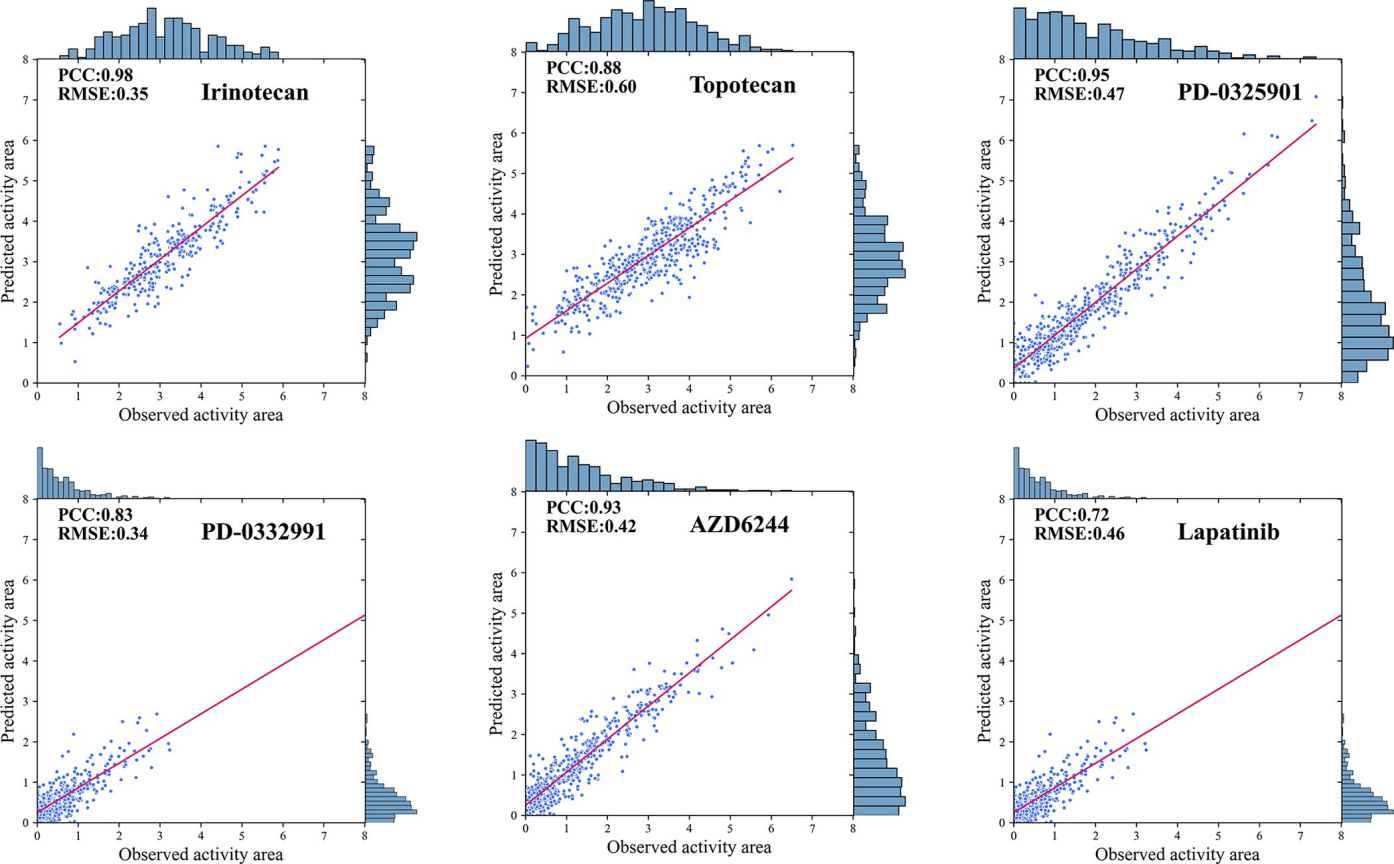
Scatter-plot between the drug responses predicted by the DBDNMF model and the original responses in CCLE.

Drugs in CCLE datasets are essential for treating cancer, and DBDNMFGER could more precisely predict the responses. In the CCLE dataset, six representative drugs, Irinotecan, Topotecan, PD-0325901, PD-0332991, AZD6244, as well as Lapatinib are further investigated in **Fig 5**, which confirms the statistically significant correlation between the predicted and observed activity area levels, demonstrating the accuracy of DBDNMF’s prediction performance for individual drug. Additionally, the histogram shows that there is excellent agreement between the predicted and observed drug activity area distributions. **Fig 5** demonstrates that the prediction accuracy is relatively high for drugs with a relatively large response span, such as Irinotecan, PD-0325901, AZD6244, and Topotecan. Regarding PD-0325901 and Lapatinib, the prediction performance is less than satisfactory and the response values are generally low, ranging from 0 to 4. Consequently, our future work will consider it during data preprocessing.

### Blind test on GDSC

The rows and columns represent the cell lines and the drugs, respectively, in the cell line-drug response matrix. In the cold-start test, we suppose a row or a column in the response matrix as the testing set, with the remaining rows and columns serving as the training set. The experiment aims to assess the ability to predict responses for a new drug or a new cell line. Tables **3** and **4** report the comparison of the ave_PCC (ave_PCC_sr), and ave_RMSE (ave_RMSE_sr) of DBDNMF and baselines for cold-start scenarios on both CCLE and GDSC datasets.

**Table 3:** The ave_PCC (ave_PCC_sr), ave_RMSE (ave_RMSE_sr) of NeuMF vs. DBDNMF for cold-start scenarios on CCLE dataset.

Datasets		ave_PCC_sr	ave_RMSE_sr	ave_PCC	ave_RMSE
NeuMF	new cell-line	0.77(±0.22)	1.42(±0.68)	0.69(±0.24)	1.34(±0.52)
	new drug	0.77(±0.23)	1.43(±0.69)	0.68(±0.24)	1.34(±0.53)
DBDNMF	new cell-line	**0.84(±0.14)**	**1.14(±0.59)**	**0.77(±0.16)**	**1.01(±0.40)**
	new drug	**0.83(±0.14)**	**1.14(±0.60)**	**0.76(±0.17)**	**1.02(±0.41)**

**Table 4:** The ave_PCC (ave_PCC_sr), ave_RMSE (ave_RMSE_sr) of TGSA, NeuMF vs. DBDNMF for cold-start scenarios on GDSC dataset.

Datasets		ave_PCC_sr	ave_RMSE_sr	ave_PCC	ave_RMSE
TGSA	new cell-line	0.66(±0.14)	1.69(±0.42)	0.56(±0.15)	1.32(±0.25)
	new drug	0.56(±0.19)	2.24(±1.18)	0.46(±0.20)	2.07(±1.19)
NeuMF	new cell-line	0.70(±0.11)	0.89(±0.30)	0.62(±0.11)	0.73(±0.23)
	new drug	0.69(±0.12)	0.89(±0.33)	0.61(±0.12)	0.77(±0.24)
DBDNMF	new cell-line	**0.72(±0.11)**	**0.79(±0.29)**	**0.63(±0.11)**	**0.63(±0.23)**
	new drug	**0.71(±0.11)**	**0.79(±0.30)**	**0.63(±0.11)**	**0.64(±0.24)**

The experiment results, as presented in Tables **3** and **4**, demonstrate that, in both leave-cell-line-out and leave-drug-out scenarios, DBDNMF outperforms other methods on the CCLE and GDSC datasets. The algorithms typically only generate features from side information, such as drug fingerprints or gene expression, in the absence of known associations between new drugs and cell lines or new cell lines and drugs. However, given some drugs or some cell lines might share similar characteristics, DBDNMF could still predict drug responses to cell lines in the cold-start scenarios.

### Drug response predictions on TCGA

**Fig 6:**
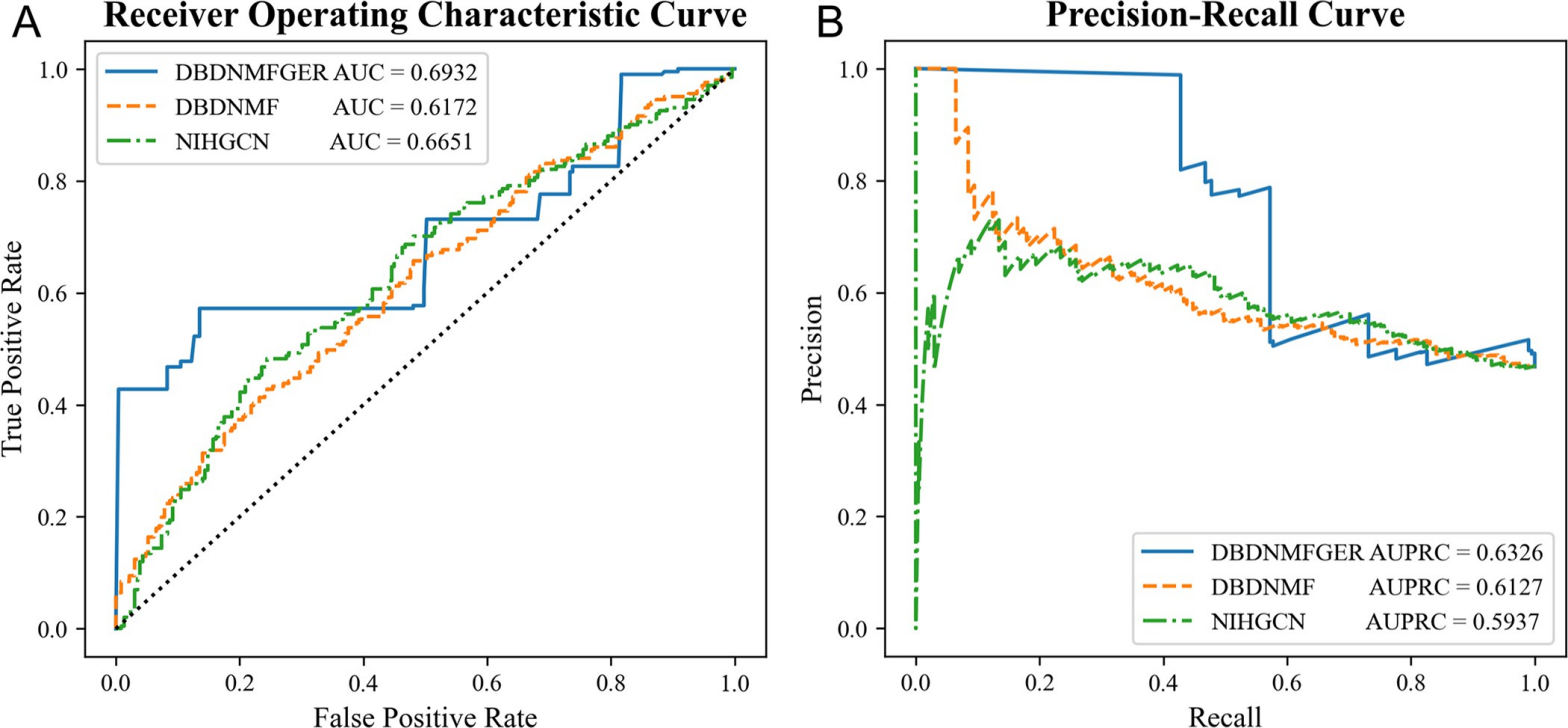
The comparison of drug response prediction. A) The Receiver Operating Characteristic Curve; B) The Precision-Recall Curve.

The drug response prediction model developed in cell line experiments (*in vitro*) is difficult to apply to real-world clinical settings (*in vivo*). The Cancer Genome Atlas (TCGA) is a comprehensive reference database for cancer research that compiles a variety of clinical data related to cancer. In the test, we used the responses from the GDSC dataset to train DBDNMF and NIHGCN, which we then applied to the TCGA dataset to predict the responses. **Table 5** displays the Area Under Curve (AUC) and the Area Under the Precision-Recall Curve (AUPRC) of the methods in terms of predicting 430 responses from 403 patients to 228 drugs from the TCGA dataset for performance comparison. Since the considered responses are not split into positive and negative classes under absolutely balanced scenarios, AUPRC may be a better metric for performance validation. As illustrated in **Fig 6**, DBDNMFGER still outperforms NIHGCN. These outcomes validate that DBDNMFGER is transferable from *in vitro* cell lines to *in vivo* datasets.

**Table 5:** Results of drug response prediction on the TCGA dataset.

Methods	AUC	AUPRC
NIHGCN	0.6651±6×10–3	0.5937±6×10–3
DBDNMF	0.6172±1×10–3	0.6127±1×10–3
**DBDNMFGER**	**0.6932±1×10–3**	**0.6326±1×10–3**

### PI3K pathway analysis on GDSC

The Phosphoinositide 3-kinase (PI3K) signaling pathway is well known for its role in regulating a wide range of cellular processes, including proliferation, growth, and apoptosis, as well as cytoskeletal rearrangement under some circumstances [21]. According to research on cancer, PI3K and other kinases in the PI3K pathway are amenable to pharmacological intervention, which makes the PI3K pathway one of the most alluring targets for therapeutic intervention in human cancer [22]. Therefore, it is of biological significance to evaluate the prediction of drugs whose target genes participate in the PI3K pathway [23]. Additionally, assessing the performance of drug predictions whose target genes participate in the ERK pathway, whose role in cellular regulation is also well known, is of great biological significance. The PCC_sr and RMSE_sr of the drugs by different methods are compared, as shown in S1A and S1B Fig.

**Fig 7:**
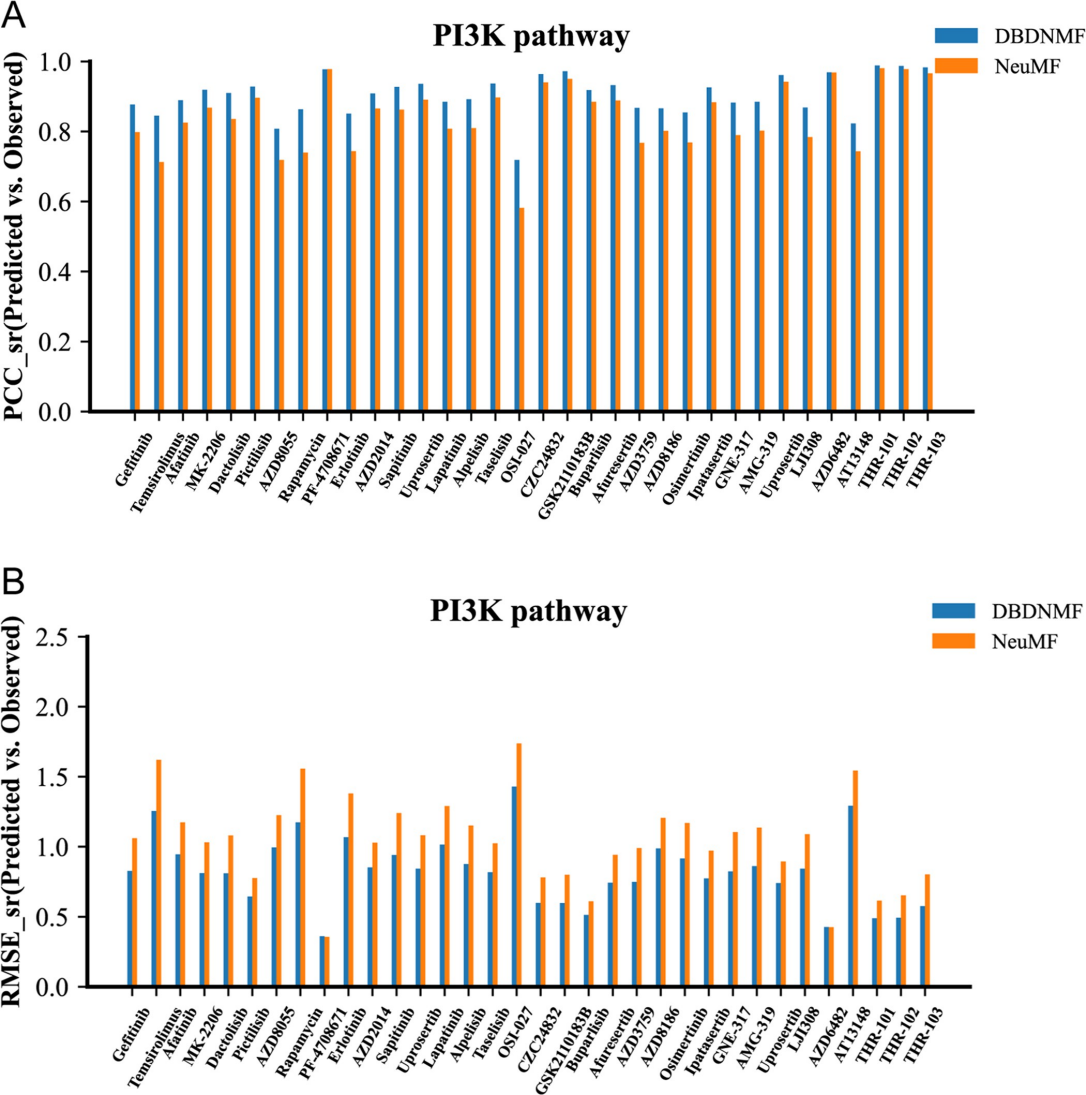
Comparison histogram of PCC_sr and RMSE_sr for the drug targets that are involved in the PI3K pathway of GDSC. **A)** Comparison histogram of PCC_sr for drugs targeting the PI3K pathway; **B)** Comparison histogram of RMSE_sr for drugs targeting the PI3K pathway.

34 drugs whose targets are involved in the PI3K pathway are chosen from the GDSC dataset to further evaluate the prediction performance of DBDNMF on individual drugs. The prediction results of the 34 drugs by various models are then compared, as shown in **Fig 7**. For most drugs targeting the PI3K pathway, DBDNMF could achieve higher PCC_sr and lower RMSE_sr than NeuMF. It conclusively demonstrates that the DBDNMF has better prediction performance when compared to other approaches. Temsirolimus, Rapamycin, and OSI-027 significantly improve the prediction effect among drugs. Temsirolimus is a potent and specific inhibitor of the mammalian target of rapamycin (mTOR) kinase which makes it an appropriate therapeutic target against tumors [24]. Rapamycin has antifungal, immunosuppressive, and potent antiangiogenic properties that significantly slow the growth of tumors [25]. OSI-027, a selective and potent dual inhibitor of mTORC1 and mTORC2, effectively inhibits the proliferation of both non-engineered and engineered cancer cell lines [26].

### Mutation consistency analysis on GDSC

**Fig 8:**
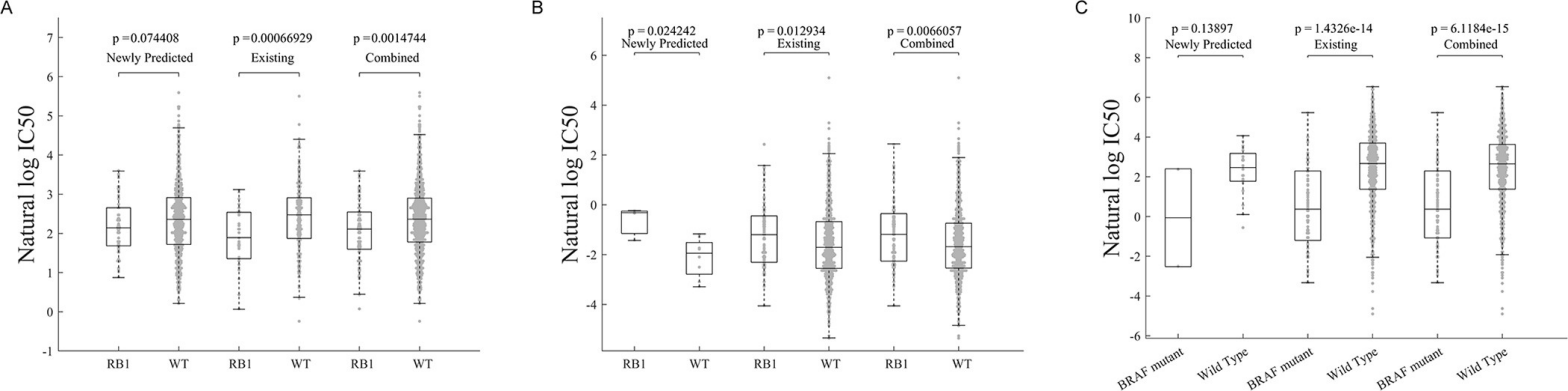
Association of drug sensitivity with cancer gene mutations is consistent for predicted and existing responses. **A)** RB1 mutated cell lines are more sensitive to the drug IOX2; **B)** RB1 mutated cell lines are more resistant to the drug Dactolisib**; C)** BARF mutated cell lines are more sensitive to the drug Refametinib.

As is known, the human retinoblastoma susceptibility gene (RB1), a tumor-suppressor gene, is mutated at various frequencies in a wide range of cancers. The RB1 protein (pRB) plays important roles in different molecular processes, such as gene transcription, DNA replication, and DNA repair [27]. IOX2, a well-characterized and selective inhibitor of the HIF prolyl-hydroxylases, has been reported to exert an anti-proliferative in human breast cancer cell lines [28]. Dactolisib, an orally administered potent dual inhibitor of PI3K/mTOR, shows promising anti-tumor efficacy [29]. IOX2’s responses have missing values in 76% of cases (743/969) and the response values of Dactolisib are missing in 1.1% of cases (14/969) in GDSC. **Fig 8A** and **8B** display the distribution of the observed and predicted responses of IOX2 and Dactolisib on RB1-mutant/wild cell lines, respectively. Based on known and predicted responses, RB1-mutant cell lines are more sensitive to IOX2, while RB1-mutant cell lines are more resistant to Dactolisib. Refametinib is a potent, allosteric MEK1/2 inhibitor that has shown promise in the treatment of a variety of tumor types, including colorectal cancer and hepatocellular carcinoma, and MEK inhibitors outperformed BRAF inhibitors in the ability to inhibit BRAF-mutated melanoma cell lines in preclinical models [30]. The predicted results shown in **Fig 8C** lead to the same conclusion that the BRAF mutant cell lines are sensitive to Refametinib.

### Consistency analysis of Top-5 sensitive and resistant drugs

There are cell lines of 30 specific cancer types involved in the GDSC dataset in total. The top-5 sensitive and top-5 resistant drugs for each cell line predicted by DBDNMF are further investigated (S1 and S2 Tables). For the top-5 sensitive drug list, Docetaxel, as a type of chemotherapy called a taxane, has been predicted as in the top-5 sensitive drug list for 657 out of 969 cell lines (67.8%). As is known, it has been proved to stop the growth of cancer cells and other dividing cells by blocking cell division, which has been approved to be used alone or with other drugs to treat breast cancer, non-small cell lung cancer, prostate cancer, gastroesophageal junction adenocarcinoma, etc. Romidepsin, a histone deacetylase (HDAC) inhibitor for the treatment of T-cell lymphoma (TCL), has been predicted as in the top-5 sensitive drug list for 859 out of 969 cell lines (88.6%). Bortezomib, the first proteasome inhibitor to have shown anti-cancer activity in both solid and hematological malignancies, has been predicted as in the top-5 sensitive drug list for 665 out of 969 cell lines (68.6%). It is known to block the activation of nuclear factor-kappa B(NF-kB), resulting in increased apoptosis, decreased angiogenic cytokine production, and inhibition of tumor cell adhesion to stroma. It has also been approved to treat multiple myeloma as well as many other hematologic and solid tumors, most of which are involved in the GDSC dataset.

For the top-5 resistant drug list for each cell line, glutathione is a vital component of the cell antioxidant system which is involved in cellular functions and human pathology and represents a rational therapeutic target against cancer. The experiments about the drug showed that the multidrug-resistant phenotype of human breast cancer cells is related to the elevated activities of glutathione peroxidase and glutathione transferase [31].

We also conducted hierarchical clustering on predicted cell line drug response matrix and found that most drugs that are clustered together, which means they show similar response levels tend to target similar signaling pathways. For example, for the cluster 2 shown in S2 Fig, the most involved drugs, such as Ulixertinib, ERK_2440, ERK_6604, SCH772984, Selumetinib, Refametinib, PD0325901, Trametinib, and VX.11e, are targeting ERK MAPK signaling pathway. As is known, the ERK MAPK signaling pathway is known to regulate various cellular processes, such as proliferation, differentiation, survival, and gene expression. Thus, it is implicated in tumorigenesis and drug resistance in leukemia and other cancers [32,33].

**Fig 9:**
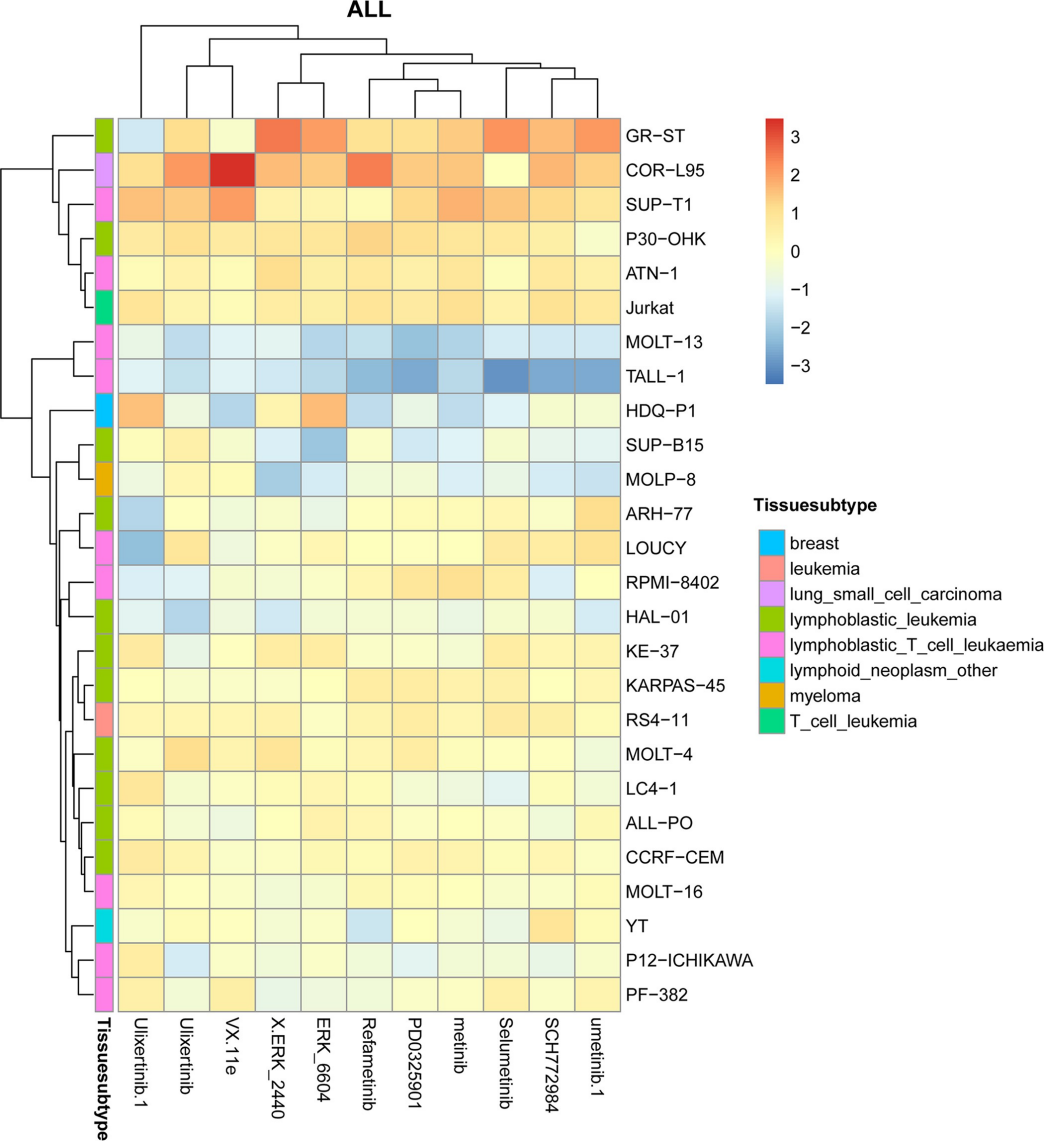
Hierarchical clustering of ALL cancer cell lines to 11 drugs come from the cluster 2.

We further investigate the response of specific cancer type-related cell lines to the above-mentioned 11 drugs from the cluster 2, and find that cell lines coming from the same tissue subtype tend to share the same pattern of response. For example, for Acute Lymphocytic Leukemia (ALL) cancer cell lines, the responses of cell lines coming from lymphoblastic_leukemia are different from those from lymphoblastic_T_cell_leukaemia, as shown in **Fig 9**.

**Fig 10:**
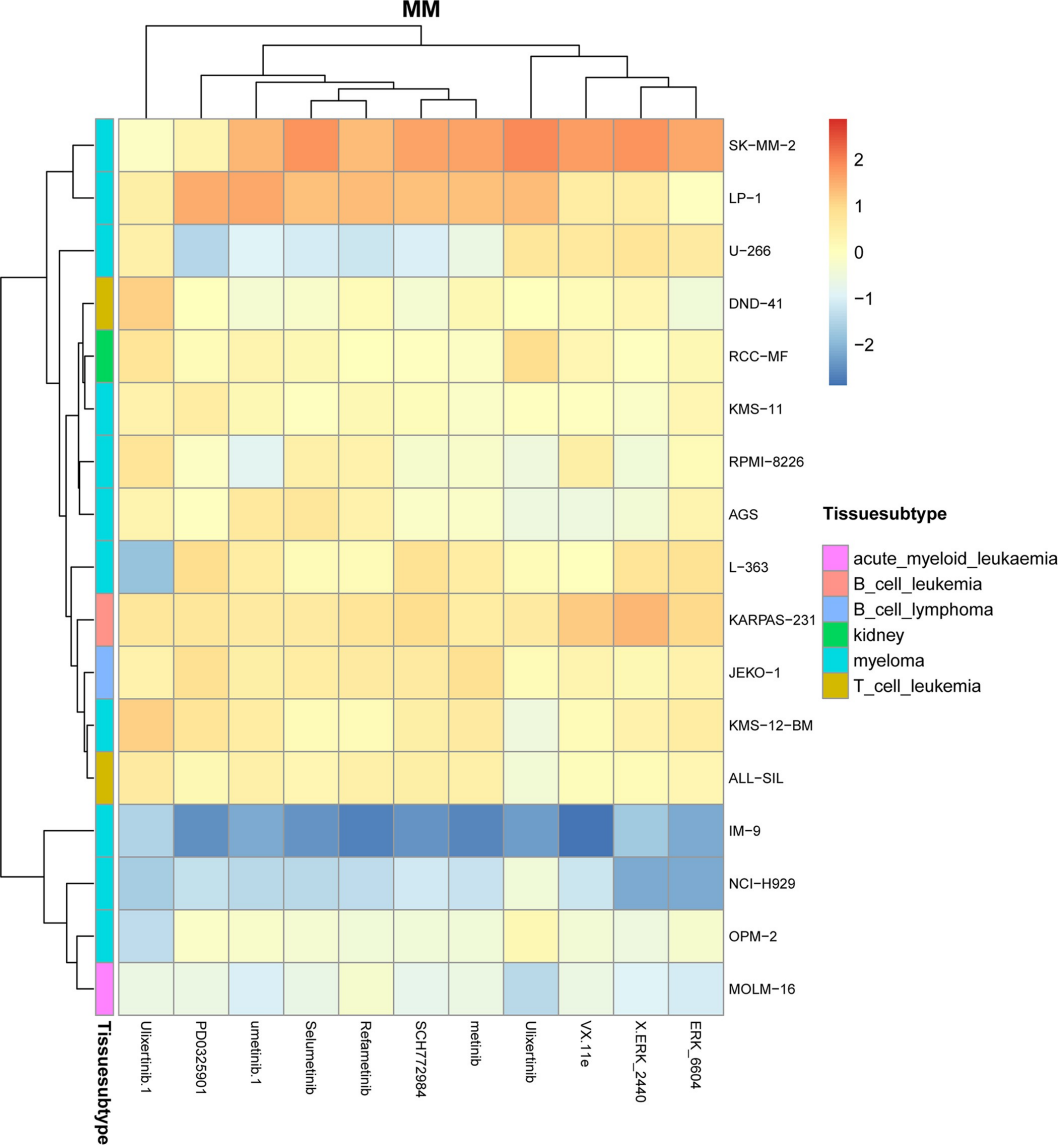
Hierarchical clustering of MM cancer cell lines to 11 drugs come from the cluster 2.

We can also get almost the same finding for the cell lines of Multiple Myeloma (MM) cancer, as shown in **Fig 10**. It may indicate that for the same cancer type, the response to the drug may differ from each other if the cancer cells come from different tissue subtypes.

## Discussion

In this paper, we propose a dual branch deep neural matrix factorization (DBDNMF) for drug response prediction. DBDNMF introduces the dual branches to handle both linear and nonlinear relations between drugs and cell lines to enhance the prediction performance. It can give potentially the most sensitive and resistant list of drugs for each cell line. Experimental results on CCLE and GDSC datasets demonstrate that the proposed method outperforms the state-of-the-art methods, and shows better biological interpretability.

Compared with conventional methods, DBDNMF can predict the drug response to explore the latent features and association mechanisms without the aid of auxiliary side information or prior hypotheses. DBDNMF constructs networks based on drug and cell lines respectively, and introduces Global Effect Removal to obtain residual which represents interaction effect.

Case studies in conjunction with biological data prove the superiority of DBDNMF over previous methods in ERK pathway prediction; the predicted and the original response of an individual drug show a strong positive correlation; the consistency between the predicted response and the original response distribution of mutant/wild cell lines verifies the reliability of the model; the top-5 sensitive and top-5 resistant drugs for each cell line demonstrate the consistency with previous studies, the clustering results on the predicted response matrix are also proved that most drugs that are clustered together tend to share similar signaling pathway and cell lines originating from the same tissue subtype prefer to cluster. Therefore, DBDNMF could predict the unknown responses of cell lines and drugs, which have certain reference significance.

In spite of the above encouraging performances, the model DBDNMF proposed in this paper still shows some room for improvement in predicting drug sensitivity. DBDNMF only refers to the known response levels of cell lines to drugs. However, on the one hand, tumor phenotypes are typically associated with, copy number variation, gene expression profile, etc. on the other hand, the drug attributes can also be achieved from drug chemical structures, drug targets, side effects, etc., but all these extra information are not made use of at present. Therefore, combining the above-mentioned side information could depict the characteristics of drugs and cell lines in more detail, which may potentially improve the response prediction performance for precision medicine. Besides, the incorporation of those kinds of side effects may also help improve the cold start issue to some extent, which will also be promising. In order to advance the methodology, it is necessary to preprocess and standardize omics datasets that come from various sources and are not in the same format in a significant way. As a consequence, the task of predicting drug response becomes more challenging due to the lack of standardization. Therefore, our future work will focus on introducing and integrating biological information from multiple sources into the model for drug response prediction performance, which may better guide practical medical decision-making on patients.

## Materials and methods

### Model structure

As is known, a complete observed matrix can be denoted by low-rank unknown latent variables as shown in (6).


$$\hat{R}=f(D)+C\cdot D+\varepsilon$$ (6)

where *f*(⋅) is the projection function, ***R***∈ℝ^*m*×*n*^ denotes the observed cell line-drug response matrix with missing values, $\hat{R}\in {\mathbb{R}}^{m\times n}$denotes the cell line-drug response matrix recovered with the prediction of all entries. ***C***∈ℝ^*m*×*r*^ represents the row latent representation of the response matrix, namely the cell line feature matrix. ***D***∈ℝ^*r*×*n*^ represents the column latent representation of the response matrix, namely the drug feature matrix. *ε* is additive white Gaussian noise with standard deviation of 1. *m* is the number of drugs, and *n* is the number of cell lines. With *r* = *rank*(***R***)<*min*(*m*,*n*), it means that there is a strong correlation between rows and columns in ***R***, so it can be projected into a low-dimensional subspace, which shows that the matrix contains a lot of redundant information.

In reality, the datasets are influenced by noise, which makes it difficult to predict the exact situation by linear transformation or nonlinear transformation only. If *f*(⋅), ***C*** and ***D*** can be computed via the known entries, it is possible to achieve the recovery of ***R***, where the latent feature variables (***C*** and ***D***) are initialized randomly, and *f*(⋅) is a nonlinear function. In this way, *f*(⋅), ***C*** and ***D*** are required to be optimized through backpropagation by solving minimization problem simultaneously.

$$\underset{f,C,D}{\min}\frac{1}{2n}{\left\|M⊙(\hat{R}-\alpha f(D)-(1-\alpha )C\cdot D)\right\|}_{F}^{2}$$ (7)

where ⊙ stands for Hadamard product, ${\left\|*\right\|}_{F}^{2}$denotes the Frobenius norm, and ***M***∈ℝ^*m*×*n*^ is a binary mask matrix with the same size as $\hat{R}$, which intends to distinguish the missing entries to be completed in the original matrix. Thus, ***M***_*ij*_ = 1 if ***R***_*ij*_ is known, and 0, otherwise. For simplicity, let Ω represent the observed entries of ***R***, while Ω^*c*^ represents the unknown entries. Therefore, the task of predicting unknown values can be approached by minimizing the difference between observed matrix ***R*** and prediction matrix $\hat{R}$. The parameter *α* is introduced to reach a compromise between nonlinear structure and linear structure.

In this paper, a dual branch deep neural network structure is proposed to address the above issue, with each branch representing the nonlinear and linear term, respectively. In the nonlinear part, we define the input drug latent feature variable as ***D***, which is approximated by applying the nonlinear activation function of the hidden layers. Similarly, the weight matrix is forced to approximate the linear part by setting a linear activation function.

In a single-layer neural network, the nonlinear function *f*(⋅) in (6) is computed by nonlinear activation function and weight matrix in the nonlinear term, and the matrix ***C*** in (6) can be approximated by weight in the linear term. Therefore, each column of the prediction matrix $\hat{R}$ can be reconstructed by minimizing the following loss function.

$$\underset{d_{i},W_{c},b_{c}}{\min}\frac{1}{2n}\sum_{i=1}^{n}m_{i}⊙{\left({\hat{r}}_{i}-\sigma (W_{c}d_{i}+b_{c})-W_{c}d_{i}\right)}^{2}+\lambda (\frac{1}{2n}\sum_{i=1}^{n}{\left\|d_{i}\right\|}^{2}+\frac{1}{2}{\left\|W_{c}\right\|}_{F}^{2})$$ (8)

where ***W***_*c*_∈ℝ^*m*×*k*^ denotes the weight matrix of the network, and *b*_*c*_ denotes the bias vector. *m*_*i*_, ${\hat{r}}_{i}$, and *d*_*i*_ are the i-th column of ***M***, $\hat{R}$, and ***D*** respectively. *λ* is the regularization parameter, and *σ*(⋅) is the nonlinear activation function in the nonlinear term, e.g., Sigmoid, Tanh, and Relu. The second part in (8) is a regularizer for model complexity regularization. It has been shown that a deep neural network can generate better representations of arbitrary nonlinear relations among input entries and outperforms single-layer structures [34]. Through the deep neural network structure, the nonlinear and linear terms are approximately expressed as (9) and (10), respectively.

$$f(d)=\sigma^{\left(L+1\right)}(W_{c}^{\left(L\right)}\sigma^{\left(L\right)}(W_{c}^{\left(L\right)}(\cdots \sigma^{\left(1\right)}(W_{c}^{\left(1\right)}d+b_{c}^{\left(1\right)})\cdots )+(b_{c}^{\left(L\right)})+b_{c}^{\left(L+1\right)})$$ (9)

$$Cd=W_{c}^{\left(L\right)}(W_{c}^{\left(L\right)}(\cdots (W_{c}^{\left(1\right)}d+b_{c}^{\left(1\right)})\cdots )+b_{c}^{\left(L\right)})+b_{c}^{\left(L+1\right)})$$ (10)

where *L* denotes the number of hidden layers, *d* is a column of matrix ***D***. $W_{c}^{l}$ and $b_{c}^{l}$ denotes the weight matrix and the bias vector of the *l*-th layer, respectively. *σ*^*l*^(.) is the nonlinear activation function of the *l*-th layer in the nonlinear term, and *l* = 1,2,…,*L*+1.

**Fig 11:**
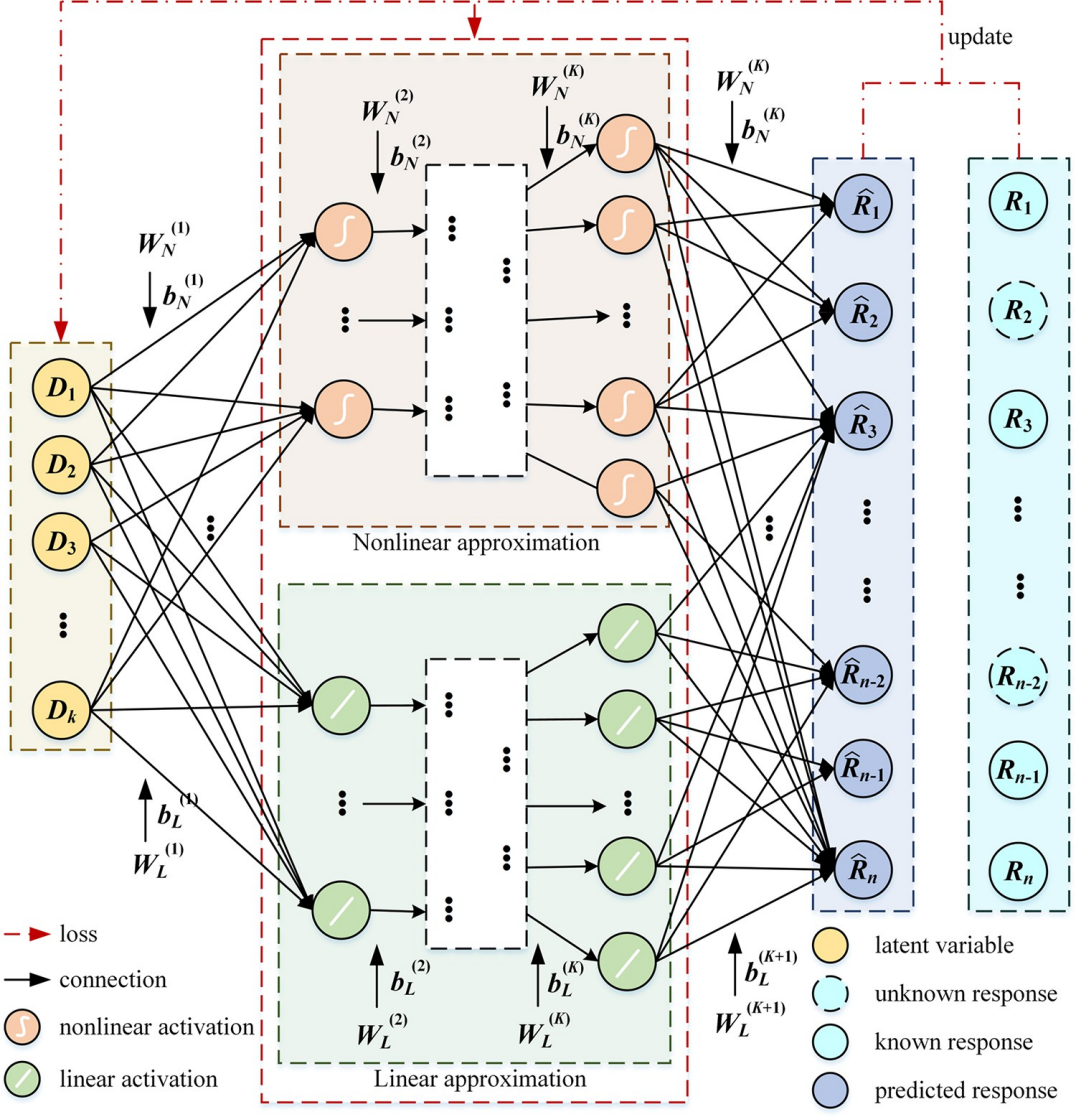
Network structure. The input layer inputs a low-dimensional unknown potential matrix. The hidden layers are divided into linear hidden layers and nonlinear hidden layers. The output layer outputs the completed cell line-drug response matrix.


**Fig 11.** depicts the structure of the proposed DBDNMF method. By solving (6), the optimized *f*(⋅), ***C*** and ***D*** can be achieved. Thus, based on (10), ***C*** is approximated by the product of the linear hidden layer weights. In a broad sense, ***C*** also represents the latent factor matrix of cell line learned in the DBDNMF.

$$C=\prod_{l=1}^{L+1}W_{c}^{l}$$ (11)

Therefore, the loss function of learning the latent representation of drugs (***D***) and the network parameters can be recast as follows by replacing (9) and (10).


$$L=L_{c}+\lambda reg_{c}$$ (12)

$$\begin{array}{l} L_{c}=\frac{1}{2\eta }\|M⊙(\hat{R}-\sigma^{\left(L+1\right)}(W_{c}^{\left(L+1\right)}\sigma^{\left(L\right)}(W_{c}^{\left(L\right)}\cdots \sigma^{\left(1\right)}(W_{c}^{\left(1\right)}D+B_{c}^{\left(1\right)})\cdots )+B_{c}^{\left(L\right)})+B_{c}^{\left(L+1\right)})\\ \;-W_{c}^{\left(L+1\right)}(W_{c}^{\left(L\right)}(\cdots (W_{c}^{\left(1\right)}D+B_{c}^{\left(1\right)})\cdots )+B_{c}^{\left(L\right)})+B_{c}^{\left(L+1\right)}){\|}_{F}^{2} \end{array}$$ (13)

$$reg_{c}=\frac{1}{2n}{\left\|D\right\|}_{F}^{2}+\frac{1}{2}\sum_{l=1}^{L+1}{\left\|W_{c}^{l}\right\|}_{F}^{2}$$ (14)

In this way, the reconstructed $\hat{R}$ can be optimized by minimizing the loss defined in formula (12), which is comprised of reconstruction error and penalty term for regularization. Previously, the prediction matrix should be pre-assumed to be a nonlinear (or linear) adaptation of the low-dimensional latent representation. However, DBDNMF does not specify whether the link between latent factors is linear or nonlinear, but both linear and nonlinear characteristics can be portrayed from the data in the final. Thus, the missing values in the original drug-cell line response matrix ***R*** can be obtained by Formula (15).

$${\hat{R}}_{i,j}={\left[\alpha f(D)+(1-\alpha )C\cdot D\right]}_{i,j},(i,j)\in \Omega^{c}$$ (15)

Similarly, the network based on cell lines can also recover the cell line-drug response matrix based on unknown cell line latent factor ***C***. The latent representation of drugs (***D***) in the network can be approximated as the product of network weights in the linear term, as shown in (16).

$$D^{T}=\prod_{l=1}^{L+1}W_{d}^{l}$$ (16)

where (⋅)^*T*^ denotes the transposition of the matrix, ***D***^*T*^ represents the drug latent factor matrix learned in the deep neural matrix factorization network in a generalized sense. In the network based on latent factor of cell lines, the loss function is defined as follows.

$$L=L_{d}+\lambda reg_{d}$$ (17)

$$\begin{array}{l} L_{d}=\frac{1}{2\eta }\|M^{T}⊙({\hat{R}}^{T}-\sigma^{\left(L+1\right)}(W_{d}^{\left(L+1\right)}\sigma^{\left(L\right)}(W_{d}^{\left(L\right)}\cdots \sigma^{\left(1\right)}(W_{d}^{\left(1\right)}C^{T}+B_{d}^{\left(1\right)})\cdots )+B_{d}^{\left(L\right)})+B_{d}^{\left(L+1\right)})\\ \;-W_{d}^{\left(L+1\right)}(W_{d}^{\left(L\right)}(\cdots (W_{d}^{\left(1\right)}C^{T}+B_{d}^{\left(1\right)})\cdots )+B_{d}^{\left(L\right)})+B_{d}^{\left(L+1\right)}){\|}_{F}^{2} \end{array}$$ (18)

$$reg_{d}=\frac{1}{2m}{\left\|C\right\|}_{F}^{2}+\frac{1}{2}\sum_{l=1}^{L+1}{\left\|W_{d}^{l}\right\|}_{F}^{2}$$ (19)

where $W_{d}^{l}$ and $B_{d}^{l}$ denote the weights matrix and bias vector of the *l*-th hidden layer respectively. Therefore, DBDNMF model differs from conventional methods in that it can accept more flexible input. In addition to learning the latent factor matrix while achieving the recovery matrix, the model is able to simultaneously combine linear and nonlinear contribution features.

### Optimization of the model

Since the loss optimization of the suggested model is non-convex, the optimal solution can prevent local minima using nonlinear convex optimization techniques like BFGS, LBFGS, iRprop+, and so on. Among these, the improved resilient back-propagation algorithm (iRprop+) is known to control the learning rate within a predetermined range without increasing the complexity of the model [35]. It can also achieve faster convergence than other neural network optimization techniques when the input of the network is unknown [36]. In light of the aforementioned facts, we choose iRprop+ for optimization in DBDNMF. Thus, the gradient of the loss function in (12) is further inferred as (20),

$$Grad_{d}=\frac{\partial L_{c}}{\partial D}+\frac{\partial L_{c}}{\partial W_{c}}+\lambda (\frac{\partial L_{reg_{c}}}{\partial D}+\frac{\partial L_{reg_{c}}}{\partial W_{c}})$$ (20)

In the same manner, the gradient of the loss function based on cell lines is shown in (21).


$$Grad_{c}=\frac{\partial L_{d}}{\partial C}+\frac{\partial L_{d}}{\partial W_{d}}+\lambda (\frac{\partial L_{reg_{d}}}{\partial C}+\frac{\partial L_{reg_{d}}}{\partial W_{d}})$$ (21)

Therefore, it is simple to compute the gradient of (12) and (17). The proposed network’s bias vector can be viewed as a column of each layer’s output, so its gradient can be calculated by also computing the gradient of the other parameters. Therefore, the parameters are initialized to Gaussian random variables following [37] and the input ***C*** or ***D*** is initialized to zero. It is also significant to select different activation functions according to different data features. In DBDNMF model, the hyperbolic tangent function is used in nonlinear approximation branch, while full connection is adopted in linear approximation branch.

## Supporting information

S1 Data Excel spreadsheet containing, in separate sheets, the underlying numerical data and statistical analysis for Figs 1, 2, 3, 4, 5, 6, 7, 8, 9, and 10. (XLSX)

S1 Fig Comparison histogram of PCC_sr and RMSE_sr for the drug targets that involved in the ERK pathway of GDSC. **A)** Comparison histogram of PCC_sr for drugs targeting the ERK pathway; **B)** Comparison histogram of RMSE_sr for drugs targeting the ERK pathway.
(TIF)

S2 Fig Hierarchical clustering of drugs based on their predicted responses to all cell lines. (TIF)

S1 Table The top-5 sensitive drugs for each cell line predicted by DBDNMF. (XLSX)

S2 Table The top-5 resistant drugs for each cell line predicted by DBDNMF. (XLSX)
