# Supplementary figures and images for: DBDNMF: A Dual Branch Deep Neural Matrix Factorization method for drug response prediction
Source: PLoS Comput Biol. 2024 Apr 4;20(4):e1012012. doi: 10.1371/journal.pcbi.1012012 (PMC11020650; doi:10.1371/journal.pcbi.1012012)

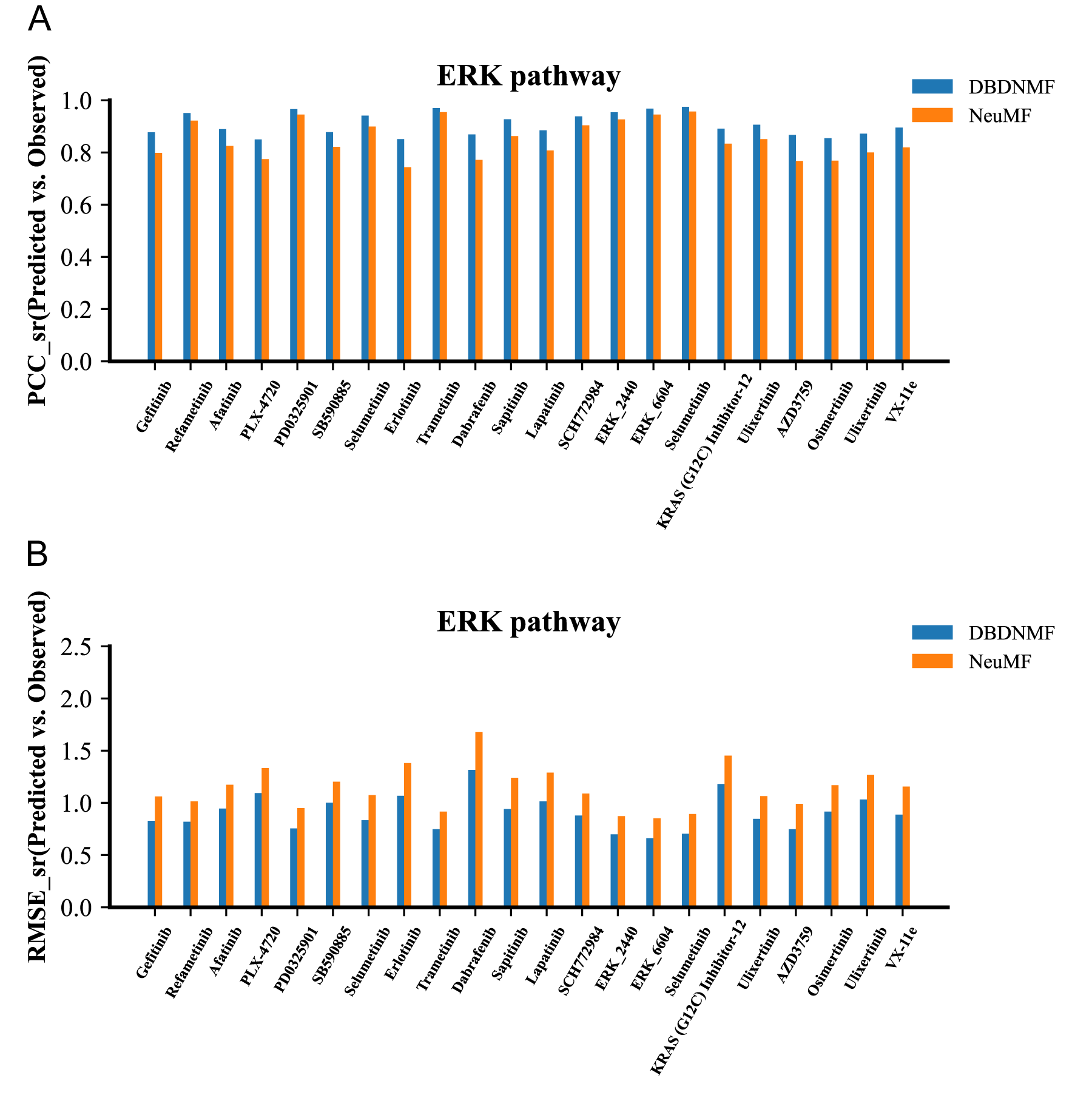

Supplement: S1 Fig — A) Comparison histogram of PCC_sr for drugs targeting the ERK pathway; B) Comparison histogram of RMSE_sr for drugs targeting the ERK pathway. (TIF) [file pcbi.1012012.s002.tif]

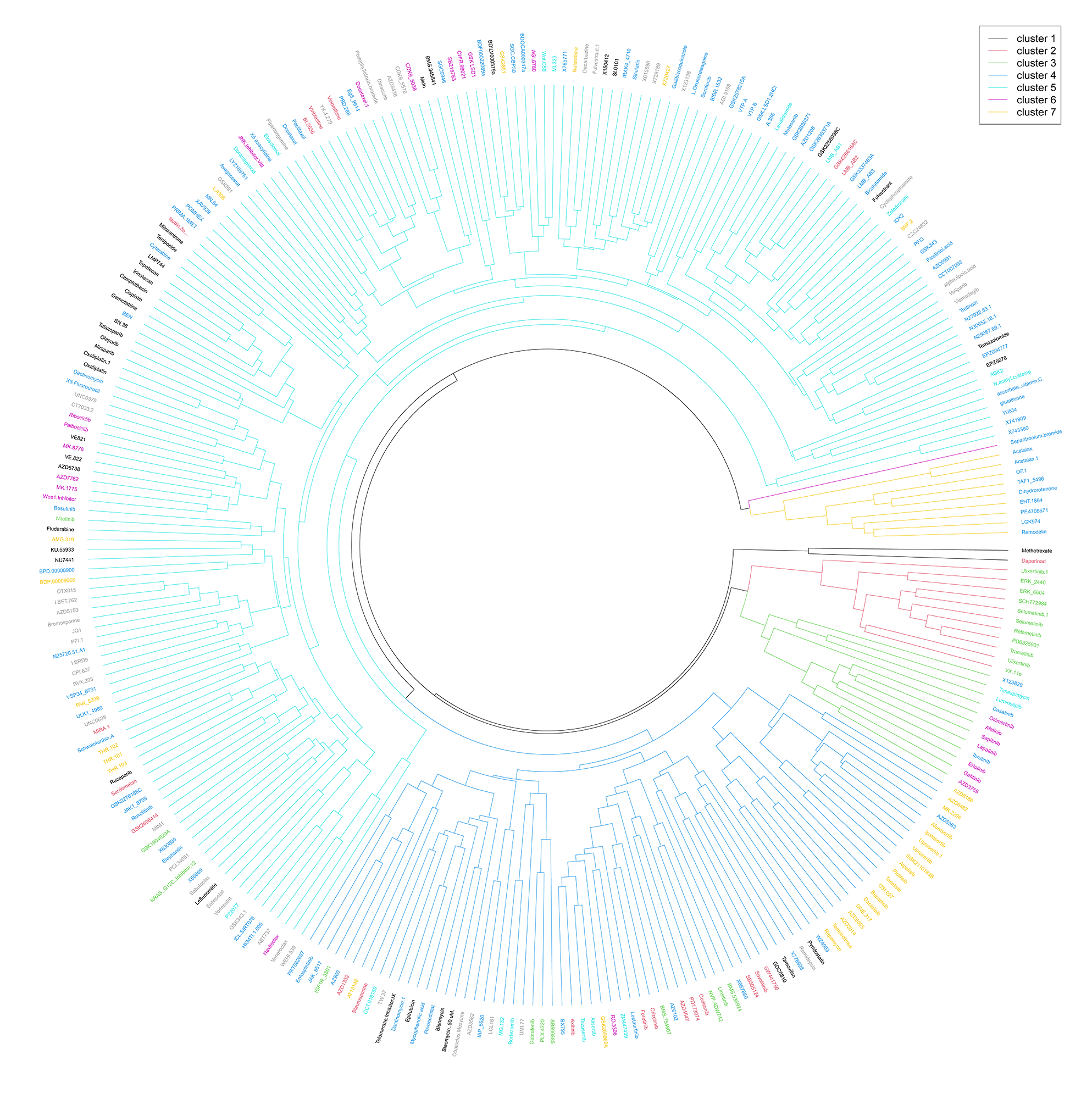

Supplement: S2 Fig — (TIF) [file pcbi.1012012.s003.tif]
